# Quantitative Analysis of Bisphenol A in Commercial Beverages

**DOI:** 10.3390/molecules31050805

**Published:** 2026-02-28

**Authors:** Ana I. Freitas, Marta S. Ferreira, José C. Marques

**Affiliations:** 1 ISOPlexis–Center for Sustainable Agriculture and Food Technology & Faculty of Exact Sciences and Engineering, University of Madeira, Campus Universitário da Penteada, 9020-105 Funchal, Portugal; jose.carlos.marques@staff.uma.pt; 2 i3N, University of Aveiro, Campus Universitário de Santiago, 3810-193 Aveiro, Portugal; 3 Department of Physics, University of Aveiro, Campus Universitário de Santiago, 3810-193 Aveiro, Portugal

**Keywords:** Bisphenol A, endocrine disrupter, food contaminants, food contact material contaminant, high-performance liquid chromatography, optical fiber sensor

## Abstract

Bisphenol A (BPA) is a widely used synthetic compound and a well-known endocrine-disrupting chemical that has been linked to a range of health issues and poses significant public health concern. Despite efforts to regulate its use in food-contact materials, BPA remains a significant food contaminant due to its widespread use and its ability to leach into consumer products. Therefore, it is paramount to continue monitoring this contaminant in the food supply chain. This work aims to assess human exposure by investigating the presence of BPA in beverages, including iced teas, fruit juices, water, and carbonated drinks. The analysis by liquid chromatography coupled with fluorescence detection reveals BPA above the limit of quantification in about 30% of samples, with concentrations ranging from 0.15 to 0.94 ng/mL. The highest detection frequencies are observed in iced teas and canned beverages, while water and glass-bottled drinks have the lowest BPA detection frequencies. In the future, we aim to use the results from this study as a reference to optimize a chitosan-coated optical fiber sensor as a possible alternative for rapid BPA detection. A preliminary test showed that the sensor can discriminate between BPA concentrations of 10–100 µg/mL in a real food matrix.

## 1. Introduction

Bisphenol A (BPA), or 4,4′-(propane-2,2-diyl)diphenol, is a synthetic compound widely used in the production of epoxy resins and polycarbonate plastics, which are then used in the manufacturing of a wide range of everyday-use consumer products, including food packaging. Epoxy resins are commonly used in the lining of metal can, while polycarbonate plastics are used in plastic food containers. Although it can result from environmental contamination [1], the presence of BPA in foods mostly results from leaching from packaging and has become a well-known food contact material contaminant [2].

Bisphenol A is classified as an endocrine-disrupting chemical, with the ability to mimic the effects of estrogen [3]. Several pathologies have been linked to BPA exposure, namely increased risk of metabolic disorders like obesity and diabetes, reproductive disorders, premature puberty, neurodevelopmental disorders and hormone-dependent cancers [3,4,5]. Due to the health risks potentially associated with BPA exposure, particularly to infants, the European Union (EU) restricted the use of BPA in plastic baby bottles [6]. In 2022, a re-evaluation of the health risks of BPA led the European Food Safety Authority (EFSA) to drastically decrease its tolerable daily intake (TDI) recommendation from 4 μg/kg body weight per day to 0.2 ng/kg body weight per day [7]. Subsequently, in December 2024, the EU banned BPA from being used in food contact materials [8].

In a bid to assess food safety and human exposure, extensive research has been conducted on the migration and leaching behavior of BPA from food packaging and food contact materials in general [2,9,10]. Equally, the determination of BPA levels in foods has also been the target of numerous studies. Given the use of BPA in the production of epoxy resins, most of these studies focus on the analysis of canned goods like vegetables, fruit, meat and fish, with reports of concentrations that in some cases exceed the EFSA recommended TDI [11,12,13]. Many of these studies also screen for BPA in beverages, particularly water, juices and carbonated drinks, packaged in different materials, with considerable variability in the BPA levels reported [2,14,15].

The trace amounts in which BPA is usually found, and the complexity of food and biological samples make the analysis of this compound very challenging and great focus has been placed on the development and optimization of methodologies for BPA analysis [16]. Traditionally, the quantitative analysis of BPA is most commonly achieved through an extraction procedure followed by gas chromatography coupled with mass spectroscopy detection or liquid chromatography coupled with photodiode, electrochemical, fluorescence or mass spectroscopy detection [17]. Recently, however, several studies exploring other approaches for a simple and quick analysis of BPA have been published, often focused on electrochemical [18,19] or colorimetric [20] sensors. Due to their advantageous characteristics, optical fiber sensors incorporating aptamers, nanomaterials or molecularly imprinted polymers have also been reported for the sensitive detection of BPA [21,22,23].

Considering the health risks associated with BPA and the recent legislation aiming to limit its presence in food products, the primary goal of this work is to investigate BPA levels across a broad range of beverages commercially available in Portugal. To this end, high-performance liquid chromatography (HPLC) coupled with fluorescence detection (FLD) was employed to analyze fruit juices, bottled waters, iced teas, and carbonated beverages from various brands.

In the future, we intend to use the data collected in this screening as a reference for optimizing a chitosan-coated optical fiber sensor for the detection of BPA in food matrices. To this end, a preliminary assay to evaluate the ability of a previously developed sensor to discriminate between different concentrations of BPA in a real food sample is also described.

## 2. Results and Discussion

### 2.1. Method Optimization and Performance

**Figure 1:**
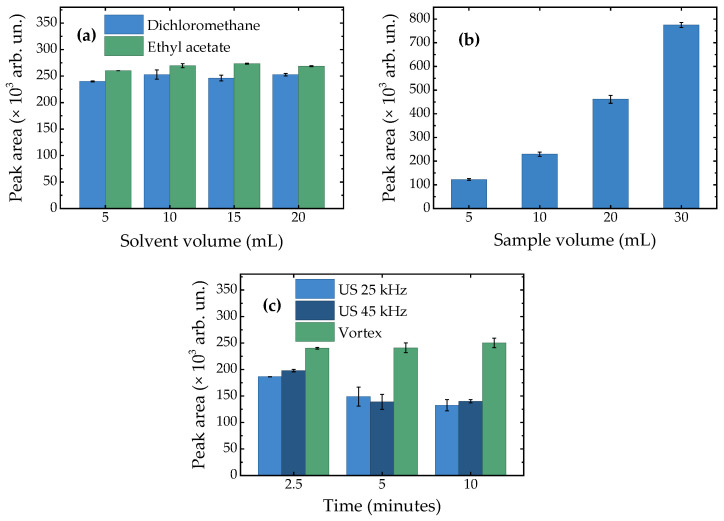
Average chromatographic peak area (×10^3^ arb. un.) obtained for each method optimization parameter evaluated: (**a**) comparison between dichloromethane and ethyl acetate, at different volumes (mL); (**b**) effect of sample volume (mL); (**c**) comparison between vortex- and ultrasound-assisted extraction (25 and 45 kHz), at different extraction times (minutes).

The efficiency of the extraction procedure was optimized by systematically evaluating five key parameters: extraction solvent, solvent volume, sample volume, extraction technique (vortex or ultrasound), and extraction time. The selection criterion for optimal efficiency was the BPA peak area. The results of the optimization assays, expressed as the average BPA peak area, are shown in Figure 1.

The extraction efficiency was initially evaluated by comparing dichloromethane and ethyl acetate as extraction solvents. For each solvent, volumes of 5, 10, 15, and 20 mL were tested, while keeping the sample volume constant at 10 mL. Ethyl acetate consistently outperformed dichloromethane, resulting in 6–10% higher peak areas across all volumes tested. Regarding solvent volume, volumes of 10, 15, and 20 mL of ethyl acetate showed very similar BPA peak areas. While 5 mL of ethyl acetate resulted in only a 4% decrease in efficiency, which represents a minimal decrease that can be balanced against the current emphasis on developing greener methods that use minimal organic solvents, 10 mL of ethyl acetate was ultimately selected for subsequent experiments. This larger volume was chosen to accommodate potential variations in emulsion volume that may arise from different matrices, ensuring a more consistent extraction performance.

Sample volumes of 5, 10, 20, and 30 mL were then evaluated. In this case, higher sample volumes led to greater BPA peak areas. Specifically, a 30 mL sample volume improved extraction efficiency by approximately 40% compared to a 20 mL sample volume.

To enhance the interaction between the sample and solvent, vortex- and ultrasound-assisted extractions (using 25 kHz and 45 kHz frequencies) were compared over 2.5, 5, and 10 min periods. Both ultrasound frequencies yielded similar BPA peak areas, which decreased for longer ultrasound exposure times. This decrease coincided with a temperature increase of approximately 5 °C for 45 kHz ultrasound and 10 °C for 25 kHz ultrasound. Considering its thermal stability, these temperature increases are not expected to cause BPA degradation. While using a glass container may enhance cavitation efficiency, both vortex- and ultrasound-assisted extractions were performed in identical Falcon tubes to ensure direct comparability between the two techniques. In this case, vigorous shaking during the vortex-assisted extraction facilitated mixing of the aqueous and organic phases, greatly promoting BPA extraction. Vortexing resulted in approximately 18% higher peak areas compared to the ultrasound-assisted methods. In this case, longer exposure times did not further improve extraction efficiency.

Regarding the validation results, detailed in Table 1, the method demonstrated good analytical performance across several key parameters. Linearity was excellent, with an R^2^ value exceeding 0.99, over a broad concentration range of 0.1 to 500 ng/mL. Accuracy, assessed through recovery experiments at low, medium, and high concentration levels (0.5, 10, and 250 ng/mL, respectively), yielded recoveries of 100.1%, 77.1%, and 69.8%, with an overall average recovery of 82%. Furthermore, the method exhibited good precision, with intra-day variations ranging from 1.6 to 8.7% and inter-day variations from 3.4 to 13.1%, across the three concentration levels evaluated. The method limit of detection (LOD), determined considering a signal-to-noise ratio of 3 (S/N = 3), was 0.05 ng/mL, and the limit of quantification (LOQ), estimated based on a signal-to-noise ratio of 10 (S/N = 10), was 0.14 ng/mL.

### 2.2. Quantitative Analysis of BPA

**Figure 2:**
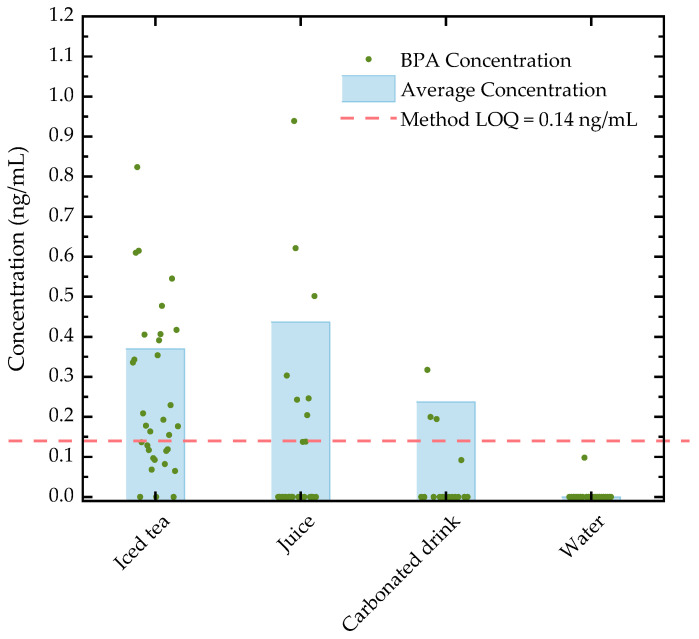
Individual and average concentrations of BPA (ng/mL) by sample type.

The optimized and validated method was used to investigate the presence of BPA in 98 beverage samples sold in Portuguese supermarkets. Bisphenol A was detected in 43 samples, with a detection frequency of 44%. Of these, 29 samples (about 30%) were above the method LOQ. The individual and average BPA concentrations by sample type are shown in Figure 2 and are also included in Supplementary Material (Table S1). Iced teas and tisanes had the highest number of samples with quantifiable amounts of BPA (59.4%), with concentrations between 0.15 and 0.82 ng/mL. In fruit juices, BPA was found in 33% of samples, of which 25.9% were above the LOQ, with concentrations ranging from 0.20 to 0.94 ng/mL. Juices had an average BPA concentration of 0.44 ng/mL, slightly higher than those in iced teas (0.37 ng/mL) and carbonated drinks (0.24 ng/mL). Bisphenol A was detected in 25% of carbonated drink samples, but quantifiable levels were found in only 18.8% of samples, ranging between 0.19 and 0.32 ng/mL. In water, BPA was detected in only one sample, packaged in a glass bottle, but in concentrations below the LOQ. Since glass bottles do not contain BPA, the presence of this compound in the sample could be due to leaching from the metal lid. Similar to metal cans, the interior of metal lids used with glass bottles is usually coated with a resin that can be a potential source of BPA [24].

Overall, the BPA concentrations found in this study for different types of beverages are comparable to those reported in the literature for similar sample types. For carbonated drinks, our results are consistent with those reported by [25,26], who found BPA at levels up to 0.607 ng/mL. In juices, ref. [27] reported concentrations ranging from 0.47 to 0.75 ng/g, comparable to those observed in this study. Little information was found for BPA levels in iced teas. However, the available studies reported BPA levels ranging from 0.075 to 0.88 ng/mL [28,29], similar to our findings. For bottled water, most studies report either non-detectable or very low BPA concentrations [29,30,31,32], although isolated cases of higher levels, up to 41.19 ng/mL, have been observed [33].

It is important to note that there is considerable variability in BPA levels reported for beverages. This variability could be attributed to several factors, such as production and storage practices, contamination in the food chain, packaging materials, limitations in the methodology for BPA analysis, or even how samples are categorized.

In terms of packaging type, except for the water sample previously mentioned, BPA was not detected in any other beverage packaged in glass bottles. On the other hand, around 58% of samples packaged in metal cans contained BPA, with concentrations ranging from 0.15 to 0.42 ng/mL. This result is consistent with the premise that BPA is present in resins used for the inner lining of metal cans, which are a known source of BPA contamination [2,34]. As for other packaging types, BPA was found in 28.6% of samples in carton packaging (TetraPak and TetraBrik), in concentrations from 0.18 to 0.94 ng/mL, and in 18.2% of samples in plastic bottles, with concentrations ranging from 0.15 to 0.42 ng/mL. TetraPak carton packaging is composed of paperboard, aluminum, and plastic and is advertised as BPA-free; however, BPA has previously been detected in products packaged in this material [35]. Similarly, BPA has also been reported in beverages packaged in polyethylene terephthalate (PET) plastic bottles. It has been suggested that the presence of BPA in beverages packaged in this type of material could be attributed to contamination in water sources, during production and storage processes, or even due to the recycling of PET plastics [10].

The effects of sample type and packaging type on BPA concentration were analyzed using a Tobit censored regression model to account for left-censoring at the limit of quantification. Thus, the statistical analysis was performed using the method LOQ (0.14 ng/mL) as the left threshold. The model included only the main effects, with “carbonated drink” and “can” defined as the reference categories for sample type and packaging type, respectively. Due to the imbalance in the number of observations across sample and packaging categories, interaction effects between these factors were not evaluated. The results of the analysis revealed that the overall model was statistically significant (*p* < 0.001), indicating that, as a whole, sample and packaging types had a significant effect on BPA concentration. Specifically, iced tea and juice samples had significantly higher BPA concentrations than the reference group “carbonated drink” (*p* < 0.001 and *p* < 0.01, respectively). As for packaging type, BPA concentrations in samples packaged in plastic and carton were significantly lower than those in the reference group “can” (*p* < 0.001). Although the model was unable to detect significant differences (*p* > 0.05) between samples packaged in glass bottles and the reference group, this may be attributed to the nature of the data, since all results were below the method LOQ, which was used as the left-threshold.

Since iced teas had the highest BPA detection rate and given the particularities of this sample type in terms of packaging (can, carton, and plastic), brands (5 brands) and flavors (lemon, mango, and peach), the data for this sample type underwent further statistical analysis. Once again, the analysis was carried out using the Tobit model, with the LOQ as the left-threshold, and “can”, “Brand 1”, and “lemon” as the reference group. The results showed that the model was statistically significant (*p* < 0.001), suggesting that, overall, packaging type, brand, and flavor had a significant impact on BPA concentrations in iced teas. As expected, the BPA concentrations in samples packaged in carton and plastic were significantly lower (*p* < 0.001) than those from the reference group “can”. In terms of brands, the analysis revealed that Brand 2 had significantly lower (*p* < 0.001) BPA concentrations compared to the reference group “Brand 1”, while Brand 4 had significantly higher (*p* < 0.001) concentrations. On the other hand, no significant differences (*p* > 0.05) were found between BPA concentrations in mango and peach flavored iced teas compared to those in the reference group “lemon”.

Considering the EFSA recommended TDI of 0.2 ng/kg body weight per day, the maximum daily intake of BPA for an adult with an average body weight of 70 kg should not exceed 14 ng. With the smallest packaging volume being 200 mL, consumption of a full package of a beverage with BPA levels above the LOQ would surpass the TDI. These findings are particularly concerning, considering that these products are also consumed by children, who have lower body weight and are more susceptible to BPA-related risks. Additionally, these are likely not the only daily sources of BPA exposure.

### 2.3. Optical Fiber Sensor Applicability

As a preliminary step toward the future development of an alternative method for rapid BPA detection in beverages, the performance of a chitosan-coated optical fiber sensor was evaluated. To this end, an iced tea sample spiked with BPA at concentrations ranging from 10 to 100 µg/mL, in increments of 10 µg/mL, was used. The fabrication and characterization of the optical fiber sensor based on a microstructured optical fiber were previously reported in [36].

Optical fiber sensors offer several practical advantages, including real-time monitoring, remote sensing capability, high corrosion resistance, and immunity to external electromagnetic interference. Some optical fiber-based sensors for BPA detection have been proposed, incorporating fiber surface modifications with antibodies, aptamers, and molecularly imprinted polymers, which enable low limits of detection and selectivity toward the target compound [21,22,23]. To the best of the authors knowledge, chitosan has not been reported as a recognition element in this type of sensor for BPA detection. Nevertheless, chitosan is an inexpensive, biodegradable, and nontoxic natural polymer, making it an attractive candidate for this application.

**Figure 3:**
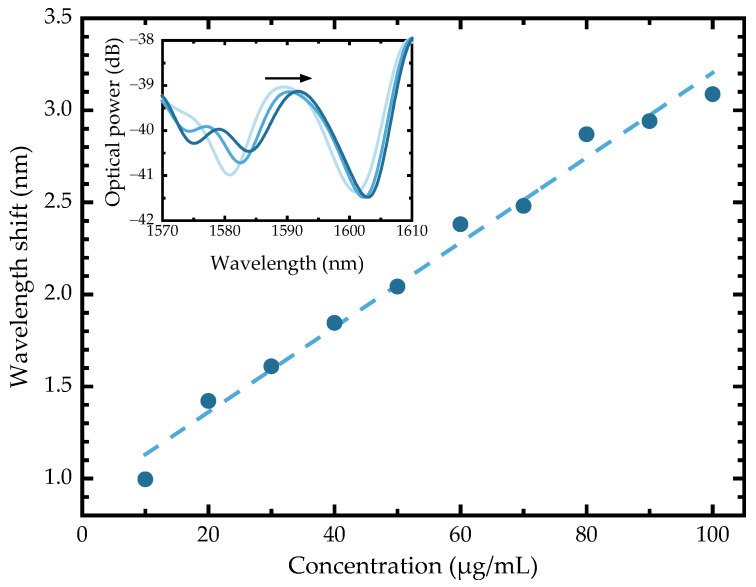
Wavelength shift with variations in concentration. Inset: filtered spectra where a wavelength shift in the peak at ~1588 nm can be observed.

The optical spectra of each BPA-spiked sample were acquired, and an appropriate band-pass filter was applied to the spectra to monitor the interferometric component susceptible to changes in the external medium. Even in a complex matrix like iced tea, increasing BPA concentration from standard addition led to an increase in refractive index, which in turn caused a shift toward longer wavelengths (red shift). This wavelength shift was measured for the filtered spectrum peak at ~1588 nm and displayed a linear behavior, as shown in Figure 3, with a sensitivity of 23 pm/(µg/mL) and a correlation coefficient of 0.986.

The current detection range of the chitosan-coated optical fiber sensor evaluated in this assay is higher than the BPA levels in beverages, which are in the ng/mL range. It is important to note that the optical fiber sensor presented in this work is at a developmental stage and is not intended as a fully optimized quantitative tool for BPA monitoring; rather, it was investigated as a preliminary proof-of-concept for its rapid detection. This study aims only to demonstrate the feasibility of using a chitosan-coated optical fiber sensor for rapid BPA detection in a complex food matrix without prior extraction or pre-concentration steps.

While the successful detection of BPA in a spiked iced tea sample confirms the sensor ability to operate in a complex matrix, further optimization is required to improve sensitivity to match the concentration levels required for BPA analysis in beverages and to provide selectivity. Nonetheless, these results provide a foundation for further optimization and represent an important step in sensor development. Further optimization to enhance selectivity and sensitivity will include, for example, chitosan modification with a crosslinker, aiming to improve its structural stability, as well as using chitosan as a molecularly imprinted polymer for selective BPA recognition.

In future work, validation parameters, including selectivity, repeatability, and long-term stability, will also need to be systematically evaluated. In particular, interference from BPA structural analogs and other polyphenols will need to be investigated, as well as inter-sensor variability.

## 3. Materials and Methods

### 3.1. Reagents and Standards

The BPA standard (≥99% purity) was obtained from Aldrich Chemicals (St. Louis, MO, USA). Stock solutions (1 mg/mL) were prepared in ethanol (≥99.8% purity), which was acquired from Honeywell|Riedel-de Haën (Seelze, Germany), while work solutions were subsequently prepared by diluting the stock solution with ultrapure water. HPLC-grade ethyl acetate and analytical-grade dichloromethane, from Honeywell|Riedel-de Haën (Seelze, Germany) and Fisher Scientific (Loughborough, Leicestershire, UK), respectively, were evaluated as extraction solvents. Acetonitrile, also HPLC-grade, was purchased from Honeywell|Riedel-de Haën (Seelze, Germany) and used as chromatographic eluent. Ultrapure water (type 1, 18 MΩ), used for the preparation of BPA solutions and as chromatographic eluent, was produced using a Millipore Simplicity^®^ UV ultrapure water apparatus (Milford, MA, USA).

### 3.2. Samples

A total of 98 samples were purchased from different supermarket chains in Madeira, Portugal. The samples were categorized as follows: water (23 samples, comprising still, sparkling and flavored water), iced tea (32 samples, including iced teas and tisanes), fruit juice (27 samples), and carbonated drink (16 samples). For each category, samples from different brands and packaged in different materials, were acquired, with volumes ranging from 200 to 750 mL. Packaging materials included cans, cartons (Tetra Pak and Tetra Brik), glass bottles, and PET plastic bottles. The number of samples by category and packaging material is described in more detail in Table 2. Samples were kept at room temperature in their original packaging until analysis and were analyzed before their “best before” date. Carbonated drinks were degassed in an ultrasound bath prior to extraction.

### 3.3. Extraction Procedure

The analysis of BPA in beverages was performed based on a liquid–liquid extraction procedure, as follows: 10 mL of ethyl acetate and 30 mL of sample were added to 50 mL falcon tubes. The mixture was vortexed for 2.5 min on a Vortex-Genie 2T mixer (Scientific Industries, Bohemia, NY, USA) and centrifuged at 10,000 rpm for 10 min, at room temperature, on a Heraeus Multifuge X1R centrifuge from Thermo Fisher Scientific (Osterode am Harz, Germany). After separation, the organic phase was transferred to conic glass vials and evaporated to dryness under a nitrogen flow. The extract was then reconstituted with 1 mL of 40% acetonitrile solution and filtered through 0.22 µm PP syringe filters (filtraTech, Saint-Jean-de-Braye, France) before chromatographic analysis. All samples were extracted in duplicate.

The extraction conditions were previously optimized by evaluating the effects of extraction solvent, solvent volume, sample volume, extraction technique (vortex- or ultrasound-assisted), and extraction time using a one-factor-at-a-time approach.

### 3.4. Chromatographic Conditions

The chromatographic analysis of BPA was performed on a Waters Alliance liquid chromatographer equipped with an auto-injector (Waters 2695 from Waters, Milford, MA, USA). Eighty microliters of extract were injected, and separation was carried out on a Waters XTerra RP18 column (4.6 × 250 mm, with 5 μm particle size; Milford, MA, USA), with the temperature set to 45 °C. A 40% acetonitrile solution was used as chromatographic eluent, at a constant flow rate of 0.5 mL/min, for a total run time of 30 min. A Multi λ Fluorescence detector (Waters 2475 from Waters, Milford, MA, USA) was used, with excitation and emission wavelengths set to 275 and 305 nm, respectively, and gain set to 10. Data was recorded and processed using the Empower Pro software, version 5.0, from Waters. Bisphenol A was identified by comparing its retention time with that of the standard, and an external standard calibration was used for quantitation purposes.

### 3.5. Evaluation of Method Performance

Method performance was evaluated in terms of linearity, accuracy, precision, and sensitivity. Linearity was assessed through a calibration curve generated from twelve solutions of different concentrations of BPA, ranging from 0.1 to 500 ng/mL. Each solution was extracted in triplicate and injected in duplicate, and the calibration curve was constructed by plotting the peak area against the corresponding theoretical concentration of each solution. The linearity was then evaluated by the coefficient of determination (R^2^) of this calibration curve.

Accuracy was determined by analyzing BPA-spiked iced tea samples at three concentration levels: 0.5, 10, and 250 ng/mL. For each concentration level, five extractions were performed, and the resulting extracts were injected in duplicate. The recovery was then calculated by comparing the measured concentration in the spiked sample to the known spiked concentration, expressed as a percentage. For the recovery assay, a calibration curve from direct injection of BPA standards was used to calculate concentrations, thereby excluding the influence of method recovery present in the calibration curve obtained from extracted standards.

Method precision was evaluated through intra-day and inter-day repeatability. Intra-day precision was determined by performing ten successive extractions of BPA-spiked samples within the same day, while inter-day precision was assessed by performing five successive extractions of BPA-spiked samples on three different days. In both cases, samples were spiked with the same BPA concentrations used in the accuracy assay. Precision was reported as the relative standard deviation (% RSD).

Finally, sensitivity was determined by calculating the LOD and LOQ. These parameters were calculated based on the linear regression analysis of the five lowest concentrations of the calibration curve.

### 3.6. Preliminary Assessment of Optical Fiber Sensor Performance

A preliminary study was conducted to assess the ability of a chitosan-coated optical fiber sensor to discriminate between different BPA concentrations in a real beverage matrix. Briefly, the sensor is composed of a 6 mm section of microstructured optical fiber (manufactured at the Leibniz Institute of Photonic Technology, Jena, Germany) spliced between two single-mode fibers (SMF28, from Corning, New York, NY, USA) and coated with three layers of a 1% (*w*/*v*) chitosan film. A detailed description of the sensor working principle, as well as the fabrication, coating and characterization procedures, can be found in the literature [36].

A store-bought iced tea was spiked with known amounts of BPA standard, in the concentration range from 0 to 100 µg/mL. The sensor was suspended horizontally and connected to a broadband light source (model ALS-CL-17-B-FA from Amonics, Beijing, China) and to an optical spectrum analyzer (OSA, Anritsu MS9740A, Kanagawa, Japan), with spectral resolution set to 0.04 nm, in a transmission setup. To perform each measurement, the sensor was submerged in 500 µL of BPA-spiked sample, placed on a microscope slide. After a 1 min contact time, the transmission spectrum was acquired between 1530 and 1610 nm.

### 3.7. Data Analysis

Data analysis was carried out in Microsoft Excel 365, while further statistical analysis was performed in RStudio Cloud (version 2025.05.1), using the AER package (version 1.2.15) to fit a censored regression model (Tobit model). The analysis was left-censored at 0.14 ng/mL to account for observations below the LOQ. OriginPro 2025 was used to analyze the spectra obtained by the optical fiber sensor.

## 4. Conclusions

This study aimed to assess BPA levels in a wide range of beverages available in the local market. The analysis of 98 samples of different types, packaged in various materials, revealed BPA in 43 samples, of which 29 exceeded the LOQ of 0.14 ng/mL, with concentrations ranging from 0.15 to 0.94 ng/mL. Although the average concentration of BPA was similar in most sample types, higher detection frequencies were observed in iced teas and in canned beverages, which is in agreement with the findings of other authors who reported that the inner lining of metal cans is a source of BPA leaching. Water samples stood out as BPA was only detected in a single sample and in concentrations below the method LOQ. While the overall low frequency of detection positively reflects the efforts made to reduce BPA exposure through food consumption, the concentrations this chemical was found in can easily lead to a BPA intake higher than the revised TDI.

To the best of the authors knowledge, this is the most comprehensive screening for BPA in beverages in the Portuguese market and represents an important indicator of food safety regarding exposure to this chemical. The recent ban on the use of BPA in food contact materials by the EU further highlights the need to continue monitoring BPA levels in our food chain. In the future, we aim to use the data collected in this study as a reference to optimize an optical fiber sensor as a possible alternative for rapid BPA screening in food products. An initial evaluation of the applicability of a chitosan-coated optical fiber sensor was conducted using a real food sample, specifically iced tea. While the sensor was able to discriminate between concentrations of 10 µg/mL, in a range from 10 to 100 µg/mL, these levels are higher than those found in beverages. Based on the BPA concentrations determined in this study, the sensor will be modified to improve sensitivity and provide selectivity, for example, through chitosan modification with crosslinkers or by using chitosan as a molecularly imprinted polymer for selective BPA recognition.

## Figures and Tables

**Table 1:** Method validation results.

Parameter	Result
Linear range	0.1–500 ng/mL
R^2^	0.995
Recovery	
0.5 ng/mL	100.1%
10 ng/mL	77.1%
250 ng/mL	69.8%
RSD (intra-day)	1.6–8.7%
RSD (inter-day)	3.4–13.1%
LOD	0.05 ng/mL
LOQ	0.14 ng/mL

**Table 2:** Information on the number of samples by beverage category and the number of samples by packaging type for each category.

Category	# of Samples	Packaging	# of Samples
Iced tea	32	Plastic bottle	9
		Carton	13
		Can	10
Juice	27	Plastic bottle	5
		Carton	15
		Glass bottle	1
		Can	6
Carbonated drink	16	Plastic bottle	6
		Can	10
Water	23	Plastic bottle	13
		Glass bottle	10

## Data Availability

Data is contained within the article and Supplementary Material.

## Supplementary Materials

The following supporting information can be downloaded at: https://www.mdpi.com/article/10.3390/molecules31050805/s1, Table S1: Sample type, packaging type, and average BPA concentration (±standard deviation) of each sample.

## Abbreviations

The following abbreviations are used in this manuscript:

BPA	Bisphenol A
EU	European Union
EFSA	European Food Safety Authority
TDI	Tolerable daily intake
HPLC	High-performance liquid chromatography
FLD	Fluorescence detection
LOD	Limit of detection
LOQ	Limit of quantification
PET	Polyethylene terephthalate
RSD	Relative standard deviation
SD	Standard deviation
OSA	Optical spectrum analyzer
