# Supplementary material for: Quantitative Analysis of Bisphenol A in Commercial Beverages
Source: Molecules. 2026 Feb 28;31(5):805. doi: 10.3390/molecules31050805 (PMC12986127; doi:10.3390/molecules31050805)
Supplement: Supplementary file 1 [file molecules-31-00805-s001.zip › molecules-4146431-supplementary.pdf]

Supplementary material

Prevalence of Bisphenol A in Beverages: Monitoring Strategies

Ana I. Freitas [1,2,3], Marta S. Ferreira [2,3,*] and José C. Marques [1,2]

1 ISOPlexis – Center for Sustainable Agriculture and Food Technology & Faculty of Exact Sciences and Engineering, University of Madeira, Campus Universitário da Penteada, 9020-105 Funchal, Portugal;
2 i3N, University of Aveiro, Campus Universitário de Santiago, 3810-193 Aveiro, Portugal;
3 Department of Physics, University of Aveiro, Campus Universitário de Santiago, 3810-193 Aveiro, Portugal
* Correspondence: marta.ferreira@ua.pt

Supplementary Table S1. Sample type, packaging type, and average BPA concentration (± standard deviation, SD) of each sample.

Sample	Sample type	Packaging type	Concentration ±SD (ng/mL)
A1	Iced tea	Plastic	0.34 ± 0.03
A2	Iced tea	Plastic	0.14 ± 0.01
A3	Iced tea	Plastic	0.21 ± 0.02
A4	Iced tea	Carton	0.41 ± 0.03
A5	Iced tea	Carton	0.18 ± 0.02
A6	Iced tea	Plastic	0.128 ± 0.009
A7	Iced tea	Plastic	0.117 ± 0.006
A8	Iced tea	Can	0.16 ± 0.01
A9	Iced tea	Carton	0.07 ± 0.01
A10	Iced tea	Carton	0.10 ± 0.01
A11	Iced tea	Carton	0.09 ± 0.02
A12	Iced tea	Carton	0.00 ± 0.00
A13	Iced tea	Can	0.35 ± 0.01
A14	Iced tea	Can	0.39 ± 0.02
A15	Iced tea	Can	0.406 ± 0.004
A16	Iced tea	Carton	0.48 ± 0.02
A17	Iced tea	Carton	0.19 ± 0.01
A18	Iced tea	Carton	0.082 ± 0.002
A19	Iced tea	Carton	0.115 ± 0.009
A20	Iced tea	Carton	0.119 ± 0.007
A21	Iced tea	Plastic	0.15 ± 0.01
A22	Iced tea	Can	0.229 ± 0.009
A23	Iced tea	Plastic	0.34 ± 0.01
A26	Iced tea	Can	0.55 ± 0.01
A27	Iced tea	Can	0.61 ± 0.05
A28	Iced tea	Can	0.82 ± 0.01
A29	Iced tea	Can	0.615 ± 0.005
A31	Juice	Carton	0.00 ± 0.00
A33	Juice	Carton	0.00 ± 0.00
A34	Juice	Carton	0.00 ± 0.00
A35	Juice	Carton	0.00 ± 0.00
A36	Juice	Carton	0.00 ± 0.00
A37	Water	Plastic	0.00 ± 0.00
A39	Water	Plastic	0.00 ± 0.00

A40	Water	Plastic	0.00 ± 0.00
A41	Water	Plastic	0.00 ± 0.00
A42	Carbonated drink	Plastic	0.00 ± 0.00
A43	Carbonated drink	Plastic	0.00 ± 0.00
A44	Carbonated drink	Can	0.317 ± 0.002
A45	Carbonated drink	Can	0.200 ± 0.006
A46	Carbonated drink	Can	0.00 ± 0.00
A48	Carbonated drink	Can	0.19 ± 0.02
A49	Carbonated drink	Can	0.00 ± 0.00
A51	Juice	Can	0.14 ± 0.04
A52	Carbonated drink	Can	0.00 ± 0.00
A53	Carbonated drink	Can	0.00 ± 0.00
A55	Carbonated drink	Can	0.00 ± 0.00
A56	Carbonated drink	Plastic	0.00 ± 0.00
A57	Carbonated drink	Can	0.00 ± 0.00
A59	Carbonated drink	Plastic	0.00 ± 0.00
A60	Carbonated drink	Can	0.09 ± 0.01
A61	Water	Glass	0.00 ± 0.00
A62	Water	Plastic	0.00 ± 0.00
A63	Water	Glass	0.00 ± 0.00
A64	Water	Glass	0.00 ± 0.00
A65	Water	Glass	0.098 ± 0.004
A67	Juice	Plastic	0.00 ± 0.00
A68	Juice	Plastic	0.303 ± 0.004
A69	Juice	Plastic	0.00 ± 0.00
A70	Juice	Plastic	0.00 ± 0.00
A71	Juice	Plastic	0.00 ± 0.00
A73	Juice	Carton	0.501 ± 0.008
A74	Juice	Carton	0.00 ± 0.00
A76	Juice	Carton	0.00 ± 0.00
A78	Juice	Carton	0.94 ± 0.01
A79	Juice	Carton	0.62 ± 0.04
A80	Juice	Carton	0.243 ± 0.002
A81	Juice	Carton	0.00 ± 0.00
A82	Juice	Carton	0.00 ± 0.00
A83	Juice	Carton	0.00 ± 0.00
A84	Juice	Carton	0.00 ± 0.00
A85	Juice	Can	0.14 ± 0.02
A86	Juice	Can	0.20 ± 0.01
A87	Juice	Can	0.25 ± 0.03
A88	Water	Plastic	0.00 ± 0.00
A89	Water	Plastic	0.00 ± 0.00
A90	Juice	Can	0.00 ± 0.00
A91	Juice	Can	0.00 ± 0.00
A92	Carbonated drink	Plastic	0.00 ± 0.00
A93	Carbonated drink	Plastic	0.00 ± 0.00
A94	Iced tea	Carton	0.00 ± 0.00
A95	Iced tea	Carton	0.065 ± 0.003
A96	Water	Plastic	0.00 ± 0.00
A97	Water	Plastic	0.00 ± 0.00
A98	Water	Plastic	0.00 ± 0.00
A99	Water	Plastic	0.00 ± 0.00
A100	Water	Plastic	0.00 ± 0.00

A101	Water	Plastic	0.00 ± 0.00
A102	Water	Glass	0.00 ± 0.00
A103	Water	Glass	0.00 ± 0.00
A104	Water	Glass	0.00 ± 0.00
A105	Iced tea	Plastic	0.42 ± 0.01
A106	Iced tea	Can	0.1764 ± 0.0004
A107	Iced tea	Plastic	0.00 ± 0.00
A108	Juice	Glass	0.00 ± 0.00
A110	Water	Glass	0.00 ± 0.00
A111	Water	Glass	0.00 ± 0.00
A112	Water	Glass	0.00 ± 0.00

Note: In samples where BPA was not detected, the concentration is shown as 0.
